# Resilience, Post-Traumatic Growth, and Transplant Effects—Gender Differences Following Liver Transplantation: A Cross-Sectional Study

**DOI:** 10.3390/healthcare13010024

**Published:** 2024-12-26

**Authors:** Víctor Fernández-Alonso, María Nieves Moro-Tejedor, Manuela Pérez-Gómez, Miriam Leñero-Cirujano, Ana María Hernández-Matías

**Affiliations:** 1 Gregorio Maranon Health Research Institute (IiSGM), 28007 Madrid, Spain; victorferal@hotmail.com (V.F.-A.); miriam.lenero@uam.es (M.L.-C.); 2 Red Cross University School of Nursing, Autonomous University of Madrid, 28003 Madrid, Spain; 3 Nursing Research Support Unit, General University Hospital, Gregorio Marañón, 28007 Madrid, Spain; 4 Liver Transplant Unit, Hospital General Universitario Gregorio Marañón, 28007 Madrid, Spain; 5 Department of Nursing, Faculty of Medicine, Autonomous University of Madrid, 28029 Madrid, Spain

**Keywords:** nursing, sex factors, coping, psychological adaptation, psychological stress, organ transplantation

## Abstract

**Background/Objectives**: The state of patients’ health following liver transplantation is influenced by a number of factors. To provide personalized nursing care, it is essential to understand the impact that the transplant has had on the patient’s life. The primary aim of this study was to examine gender differences in the overall health effect following liver transplantation. **Methods**: A cross-sectional study was conducted using the Connor–Davidson 10 Resilience Scale, the 21-item Post-Traumatic Growth Inventory, and the Transplant Effects Questionnaire. Data were collected during May–July 2021, and statistical analyses were performed. **Results**: A sample of 174 liver transplant patients who completed questionnaires were included in this study. Of these, 24.1% were women. Psychological resilience in men was correlated with personal strength, whereas in women, it was associated with relationships with others. Significant gender differences were found in psychological post-traumatic growth since liver transplantation. Additionally, resilience levels were positively and significantly associated with adherence to immunosuppressive therapy following the transplant. However, no mediating or moderating effect of resilience was identified. **Conclusions**: The results provide valuable insights for validating and improving interventions from a gender perspective. Nursing care must incorporate a gender perspective to deepen our understanding of the emotional determinants and the ability to deal with them.

## 1. Introduction

Liver transplantation (LT) has been demonstrated to markedly enhance both survival and quality of life for patients with terminal liver disease [1]. Various factors influence post-transplant health, encompassing both physical and mental well-being. Both the experience of chronic liver disease and the LT procedure can evoke feelings of distress and trauma. Indeed, studies have shown that patients often find the diagnosis and intensive care experience to be traumatic [2]. Resilience, defined as the ability to maintain focus despite adversity, is linked to social support, depression, quality of life, anxiety, cognitive ability, and age [3,4]. Nursing interventions, coordinating patient care and providing education, should aim to improve outcomes by identifying sources of motivation and coping mechanisms, such as support groups and spirituality [5,6].

Post-traumatic growth (PTG) suggests that stressful events can activate personal resources, enhancing functionality and serving as a protective factor that enables individuals to transform threats into challenges [7]. Pérez-San-Gregorio et al. (2017) found that PTG after LT is linked to quality of life, indicating its importance in ensuring long-term well-being [8]. Funuyet-Salas et al. (2019) observed differences in PTG among LT patients based on perceived health and vitality [9]. PTG benefits recipients as they recover, with support being crucial for eligibility and outcomes [10]. Higher PTG levels in LT recipients are linked to more adaptive coping strategies [11]. Pérez-San-Gregorio et al. (2018) found that adherence to treatment was positively associated with social disclosure and negatively with guilt [12].

Incorporating a gender perspective in research is good practice [13]. Race and gender impact the chances of receiving an LT [14]. Women’s health outcomes are particularly affected by gender bias in healthcare, suggesting disparities in the transplant system [15]. Melk et al. (2019) highlight gender differences in the access and outcomes of transplant recipients, with 35% women and 65% men. Social determinants of health are key to health inequities, and gender is often overlooked in studies [16]. Listabarth et al. (2022) identified disparities in access to and management of liver transplants in patients with alcohol-related liver disease [17]. The authors concluded that further research with a gender perspective is necessary to gain a more comprehensive understanding of this issue [17]. In addition, prior research has demonstrated the significance of gender in behaviors as crucial as adherence to immunosuppressive treatment [18,19,20]. The recent study by Chen et al. (2021) studied the differences in gender and roles in the experience after LT, revealing differences based on their gender and roles between the main beneficiaries and caregivers, whose claims were based on the potential influences of tradition, culture, and modern medicine [21]. The incorporation of a gender perspective has the potential to advance equity, improve health, reinforce policy development, facilitate innovative approaches, and refine solutions that address the diverse needs of society [22]. PTG represents a significant element influencing psychological well-being. Conversely, negative growth outcomes may compromise the physical and mental health of the patient, subsequently impacting adherence. Nevertheless, no studies have been identified that examine the interrelationship between general mental health, post-traumatic growth, and gender-based resilience disparities following LT.

The principal aim of this study was to analyze gender differences with regard to psychological resilience, psychological post-traumatic growth, and transplant outcomes following LT. The secondary aims were the following: (1) to examine the relationship between resilience levels and health outcomes following liver transplantation; and (2) to explore how resilience mediates and moderates the relationship between post-traumatic growth and the effects of organ transplantation in liver transplant patients.

## 2. Materials and Methods

### 2.1. Study Design

A descriptive cross-sectional single-center study was carried out at the liver transplant unit at Gregorio Marañón General University Hospital. Gregorio Marañón General University Hospital is a low–medium volume transplant center with a mean of 46.5 LTs per year [23]. The nursing process is a discrete element within the broader process map of the liver transplant unit. The nursing staff is responsible for coordinating the post-transplant consultation and for providing preventive care and health promotion services during the first year following the transplant. During the first six months, the nurse conducts weekly or biweekly checkups and subsequently monthly visits until the first year.

### 2.2. Study Participants and Setting

The inclusion criteria were adult liver transplant patients under follow-up at the liver transplant unit at the Gregorio Marañón General University Hospital, with no age limit and with the ability to understand and complete the questionnaires provided for the study. The exclusion criteria were patients who had received an organ transplant other than the liver and patients with a known history of acute psychiatric pathology. A review of clinical history was conducted, and patients who, due to the presence of a health condition, could not complete the questionnaires were also excluded. Overall, no patients were excluded due to acute psychiatric disorders, including diagnoses of schizophrenia, unstable bipolar disorder, and other active psychotic conditions. It is noteworthy that no patients with stable alcohol dependence were excluded, nor were any patients with these characteristics identified. Indeed, such exclusion criteria are not formally employed in the context of liver transplantation in Spain.

### 2.3. Data Quality Assurance

A preliminary assessment of the data collection instrument was conducted on 5% of patients at the same hospital before the actual data collection to verify the applicability of the instrument and make necessary adjustments. These samples were not included in the final analysis. The collected data were examined to ascertain their completeness, accuracy, and clarity. Incomplete data were discarded and counted as non-responses.

### 2.4. Data Collection

Data were collected between May and July 2021. All liver transplant patients under follow-up at the liver transplant service of the Gregorio Marañón Hospital (*n* = 297) were invited to participate in this study. The patients were contacted by telephone, and the purpose of this study was explained to them. Those patients who decided to participate voluntarily did so at the nurse’s office at the Gregorio Marañón General University Hospital, having previously scheduled a time for the meeting. The nurse provided the information and informed consent sheet for the participants and later hand-delivered the paper questionnaires for the participants to fill out. The participants returned the completed questionnaires to the liver transplant nurse practitioner. Sociodemographic variables were collected including age, gender, and date of LT. Multiple data collection instruments were used. Instruments were freely available. The questionnaires were provided on paper and handed out personally by the research nurse to the participants. The questionnaires were self-completed by the patients, who returned them by hand to the research nurse.

### 2.5. Study Instruments

To establish the degree of resilience, the Connor–Davidson 10 Resilience Scale (CD-RISC) was used [24], which has been validated previously in a Spanish population [25] and has also been previously used in LT patients [6]. We consider the resilience score as a health conditioner that can impact the effects of transplantation. The CD-RISC-10 is a 10-item unifactorial scale. The participant responds to each of the items (“Not at all”, “Rarely”, “Sometimes”, “Often”, “Almost always”), assigning a value between 0 and 4 to each response, respectively, and the final value is the sum of the scores of the 10 items. The cut-off value for a normal resilience is between 28 and 35 points. The CD-RISC-10’s reliability has obtained a Cronbach’s alpha of 0.81, according to a validation study in the Spanish population [26].

The evaluation of post-traumatic growth was carried out with the 21-item Post-Traumatic Growth Inventory (PTGI) scale [7]. The PTGI is a 21-item self-report measure designed for assessing positive outcomes reported by people who have experienced adverse life events including bereavement. The full-scale Cronbach’s alpha was 0.90. The factors which emerged also showed substantial internal consistency, ranging from 0.67 to 0.85: New Possibilities; Relating to Others; Personal Strength; Spiritual Change; Appreciation of Life. The PTGI is scored on a 6-point Likert scale ranging from 0 (“I did not experience this change because of my loss”) to 5 (“I experienced this change to a very great degree because of my loss”). Greater positive changes after the loss are suggested with a higher sum score of all items [7]. The Spanish version has demonstrated good psychometric properties [27].

Finally, the Transplant Effects Questionnaire (TxEQ-Spanish) is important for assessing and comparing these effects and can help optimize treatment. It consists of 22 items scored on a 5-point Likert scale ranging from “strongly agree” to “strongly disagree”. It contains five subscales that assess concerns about the transplant, guilt regarding the donor, disclosure, adherence, and responsibility (items, e.g., “I am worried about damaging my transplant”). The score for each subscale is calculated by dividing the total score by the number of items. Higher scores show a higher degree of the dimension in question. All five subscales’ Cronbach’s alpha values—worry 0.82, guilt 0.77, disclosure 0.91, adherence 0.82, and responsibility 0.83—were satisfactory as a measure of reliability [12].

The methodology employed to score the studied scales is detailed in Supplementary File S1.

### 2.6. Data Analysis

An exploratory analysis was carried out. Data analysis using descriptive statistics, means, standard deviation (SD), minima and maxima, and frequencies (as appropriate) were calculated for all variables. An exploratory analysis of the data was performed to identify outliers or extreme values and characterize differences between groups of cases. The most appropriate statistical techniques were identified, and the data were subjected to a Kolmogorov–Smirnov normality test to ascertain whether they exhibited a Gaussian distribution. Student’s *t*-test and the chi-squared test were used to compare the different variables. For those non-parametric samples, the Wilcoxon sign, Mann–Whitney U, and Kruskal–Wallis tests with time grouped into four categories were used. We performed homogeneity tests on age, time of LT, and resilience scale score that allowed us to confirm that variability between gender subpopulations did not significantly affect the interpretation of the data. The independent variables were those that showed statistical significance in the bivariate analysis or were considered relevant in the conceptual framework of this study. To assess the mediating effect of resilience between PTG and TxEQ-Spanish, we conducted the Sobel–Goodman mediation test, adjusting for the following control variables: age, years since LT, and gender. To determine the correlation between the different instruments used, the Spearman’s rho coefficient was calculated. An explanatory linear regression model was developed separately for men and women to examine the factors associated with resilience. The dependent variable was resilience, measured by the total score on the CD-RISC-10 questionnaire. The analyses were performed with STATA v.16.

### 2.7. Ethical Considerations

This study and its written consent form were approved by the Ethical Committee of Gregorio Marañón General University Hospital (code IMPACT_TH of the 02/2021 min). All research was conducted in accordance with the principles articulated in the Declaration of Helsinki [28]. Written informed consent was obtained from all participants. This study followed the STROBE criteria for quantitative studies [29]. Each participant was provided with an information page and an informed consent form.

## 3. Results

**Figure 1:**
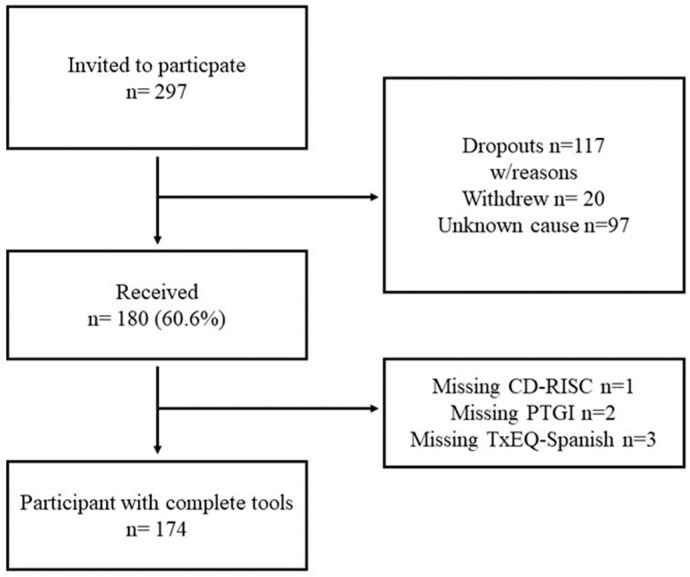
Flow chart study sample. CD-RISC: Connor–Davidson 10 Resilience Scale; TxEQ-Spanish: Transplant Effects Questionnaire Spanish; PTGI: Post-Traumatic Growth Inventory.

Of the total cohort of 297 LT patients invited to participate in this study, 180 (60.6%) returned the questionnaires. Of these, 174 (58.6%) provided complete responses to all questionnaire items (Figure 1).

Of the *n* = 174 LT patients who answered, the average study age was 61.06 (SD 11.33) years, with a minimum value of 24 and a maximum of 86 years old. The average LT age was 51.05 (SD 11.54). A total of 24.1% (N = 42) were women. The stratification of the female sample by age was 35 years old or younger N = 2 (4.8%), 36 to 49 years old N = 5 (11.9%), 50 to 64 N = 13 (31%), and 65 years old or older N = 22 (52.4%). Following the same stratification, the male sample by age was N = 5 (3.8%), N = 10 (7.6%), N = 68 (51.5%), and N = 49 (37.1%). The average number of years from LT was 10.01 years (SD 7.73) (Table 1).

The level of resilience of the participants surveyed (N = 174) was on average 30.83 (SD 6.53) considering that 44.3% (N = 77) had a normal resilience level, 27% (N = 47) had a high resilience level, and 28.7% (N = 50) had a low resilience level. Male participants obtained a median value of 32 [IQR 27–36], whereas female participants obtained a value of 32 [27,28,29,30,31,32,33,34,35]. No statistically significant relationships were found between genders (*p* = 0.525).

Regarding the PTG after LT, the results of the bivariate analysis show that women experience higher growth than men in all the inventory dimensions. This was statistically significant for the dimensions of new possibilities, spiritual change, and personal strength. Regarding our results on the effects of transplantation using the Tx-EQ-Spanish, we performed an analysis of two independent samples by gender, without finding significant differences in this regard (Table 2).

A further analysis was conducted in accordance with our secondary study aim. Our findings revealed a significant relationship between resilience across all the dimensions of the Post-Traumatic Growth Inventory and the score for adherence to immunosuppressive treatment after liver transplantation (Table 3).

### 3.1. Multivariable Analysis

A multiple linear regression analysis of resilience adjusted by gender was carried out (Table 4). In the regression model by male gender, the variable that had a specific weight in affecting resilience after liver transplantation was personal strength (R-squared = 0.189). In contrast, in the regression model by female gender, the variable with the highest specific weight in affecting resilience after liver transplantation was relationship with others, followed by adherence to immunosuppressive treatment (R-squared = 0.484).

### 3.2. Mediation and Moderation Analysis of Resilience

Mediation analysis was performed to determine whether the effect of the independent variable (PTG) on the dependent variable (TxEQ-Spanish) could be mediated by a change in the mediating variable (resilience, CD-RISC).

**Figure 2:**
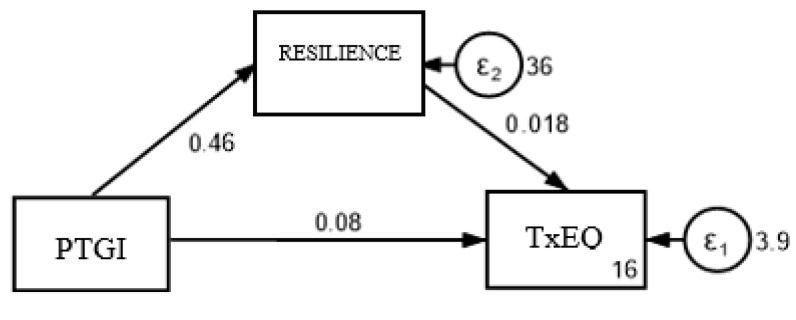
Conceptual diagram of resilience mediation between PTG and the TxEQ-Spanish.

As shown in Figure 2, the direct effect (c’) refers to the path from PTG to TxEQ-Spanish, while controlling for the mediating variable; the indirect effect (ab) refers to the effect of PTG on TxEQ-Spanish through the mediating variable, and the total effect (c) refers to the sum of the direct and indirect effects of PTG on TxEQ-Spanish. This total effect occurs when the mediating variable is excluded. The results show significant results in the direct (c’ = 0.086, *p* = 0.005) and total (c = 0.095, *p* = 0.001) effect of the PTG on the TxEQ-Spanish, as shown in Table 5. The indirect effect of the model is not significant (ab = 0.009, *p* = 0.478), since the zero value is included in the 95% confidence interval (LI = −0.015, LS = 0.033), and since the Sobel test described values that were not statistically significant for partial mediation (z = 0.789, *p* = 0.478). The proportion of the total effect that is mediated is 0.092; this indicates the proportion of the total effect that is explained through the mediating variable resilience.

**Figure 3:**
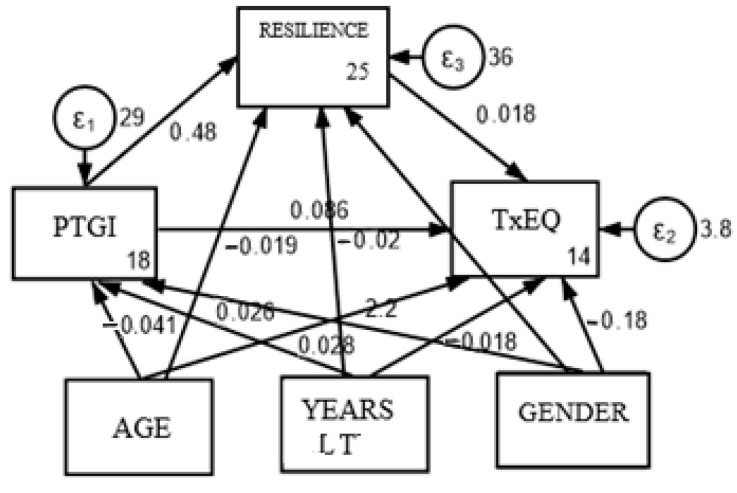
Conceptual and statistical diagram of moderated mediation.

Additionally, a moderated mediation analysis was conducted to determine whether the mediation results from the previous analysis were independent of certain contextual variables. The contextual variables examined included gender, age, and years since liver transplantation (LT). The conceptual and statistical framework for this analysis is illustrated in Figure 3. The results of the moderated mediation analysis indicated that gender (95% CI: LI = −0.882, LS = 0.534), age (95% CI: LI = −0.074, LS = 0.045; LI = −0.001, LS = 0.058), and years since LT (95% CI: LI = −0.057, LS = 0.021) were not significant moderators of the relationship between the two variables.

The results suggest that there is no significant mediated effect through the variable resilience. The proportion of the total mediated effect is 9.2%, indicating that about 9.2% of the total effect of PTG on TxEQ-Spanish is explained through resilience. The ratio of indirect to direct effect and the ratio of total to direct effect provide additional information on the relative magnitude of these effects.

**Figure 4:**
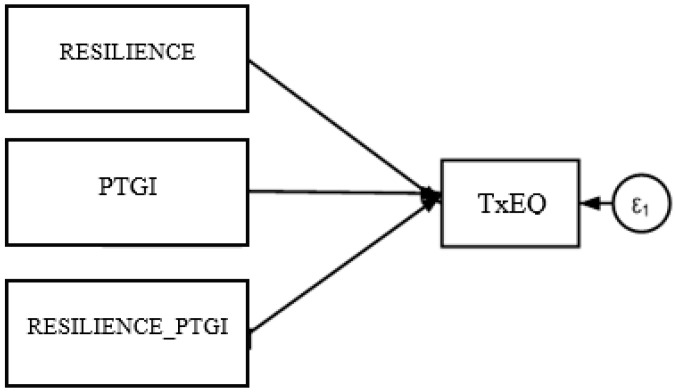
Results of the moderation analysis with resilience as a moderator.

The moderation analysis was conducted to assess whether the strength of the relationship between PTG and TxEQ-Spanish is influenced by resilience levels in liver transplant patients. As illustrated in Figure 4, the analysis considered PTG as the predictor variable, TxEQ-Spanish as the dependent variable, and resilience as the moderating variable. The results indicated that resilience does not have a moderating or modifying effect on TxEQ-Spanish (−0.001, *p* = 0.799).

## 4. Discussion

It is imperative to give due consideration to gender-based differences in the context of treatment adherence in LT patients. This approach can enhance the safety of pharmaceuticals and improve the survival rate of transplanted organs. Our results do not indicate the presence of a mediating and moderating effect of resilience on PTG, or on the psycho-emotional effects of transplantation. A better understanding of the sex- and gender-related differences in this field offers the potential for a tailored therapeutic approach and follow-up for the management of these patients.

The results demonstrate that women who have undergone LT score higher in all dimensions of post-traumatic growth (PTG), especially in “relationship with others” and “spiritual changes”. PTG significantly contributes to resilience, differing by gender: men in “personal strength” and women in “relationship with others”. Pérez-San Gregorio et al. (2017) found no gender differences, differing from our findings. Both studies found no significant differences between time since transplantation and PTG [8]. Martín-Rodríguez et al. (2018) found that those who frequently thought about their donor experienced greater PTG, especially in spiritual growth, new life appreciation, and new possibilities post-transplant [30]. Although our objective was not to analyze the impact of thinking about the donor, considering our gender-based findings, it may be beneficial to explore both factors in future studies. Our results suggest that men, who exhibited less PTG, might benefit from the psychological integration of donor thoughts to enhance PTG through increased gratitude [7].

The observed gender differences in post-traumatic growth (PTG) and resilience among liver transplant patients may be attributed to a complex interplay of biological, psychosocial, and cognitive factors. Hormonal differences, such as the effects of estrogen and testosterone, may influence stress responses and coping mechanisms, while socialization processes and gender roles shape how individuals respond to trauma. Women tend to engage more in rumination and emotional processing, which may contribute to their reported higher levels of PTG. Men, in contrast, often employ more problem-focused coping strategies, which impacts their resilience in distinct ways [31]. These findings align with broader psychological research on gender differences in trauma responses and recovery. The results of this study, which indicate that women experienced greater growth across all dimensions of the PTGI, with statistically significant differences in new possibilities, spiritual change, and personal strength, further corroborate these established patterns in the literature on gender and psychological adaptation to stress.

Our study aligns with Burra et al. (2013), whose study indicated that in order to gain a deeper understanding of gender-related factors in liver transplantation, it is necessary to move beyond a binary view, recognizing more complex processes, influenced by hormonal, social, and age factors [32]. In heart transplants, men and women exhibit different coping styles and stress responses [33]. Pérez-San-Gregorio et al. (2017) found that liver transplant patients use more adaptive coping strategies, linked to greater post-traumatic growth [11]. Our findings indicate that women score higher in all PTG dimensions, suggesting they have more effective coping strategies, a hypothesis for future studies. Another study found that men needed to enhance dignity and family roles, while women sought positive experiences and psychological support [21]. Our results highlight gender differences, underscoring the necessity for gender-specific strategies and interventions.

Tomaszek et al. (2021) found that resilience predicts PTG in kidney transplant recipients [34]. Despite gender differences in PTG in our study, we found no gender differences in resilience post-liver transplantation or in relation to time since transplantation. Fallon et al. (2020) noted that resilience pathways differ between sexes, with women experiencing more stress-related disorders [35]. Our study found resilience correlates with all dimensions of PTG and better adherence to treatment. Hence, nursing should focus on enhancing resilience in transplant patients. For men, this involves boosting personal strength and motivation. For women, it involves strengthening family trust and support groups. A study has analyzed liver transplant patients using the TxEQ-Spanish questionnaire. Tarabeih et al. (2020) studied Israeli liver transplant recipients with a mean of 7 years post-LT and a maximum age of 55 years, finding scores for worry (5), guilt (2.45), disclosure (4.65), adherence (1.25), and responsibility (3.2) [36]. Our results, with a mean patient age of 61 years and an average of 10 years since LT, differ significantly. A recent study on young adult transplant recipients noted gender differences in therapeutic adherence [37]. While our results do not show gender differences, they do reveal a negative correlation between resilience and both worry and guilt. Treatment adherence significantly correlates with resilience, particularly in women, likely influenced by the close follow-up from a liver transplant nurse practitioner at our hospital.

Finally, the study about quality of life after liver transplantation by Onghena et al. (2016) concluded that multidisciplinary interventions of psychological and biosocial treatment are needed to improve quality of life [38]. Guilt, responsibility, and worries are linked to limited mental health, while higher mental health is associated with disclosure about transplantation [39]. Our results showed a strong correlation between different scales and questionnaires. Resilience is crucial for patients undergoing liver transplantation to face possible adversities. Reducing guilt and worry and improving adherence to immunosuppressive treatment will enhance resilience. Nursing should promote disclosure and adherence to immunosuppressive treatment, guiding and empowering patients to cope with transplantation challenges. This involves focusing on personal strength for men and relationships with others for women. Despite limitations, our results highlight the need for gender-specific interventions to improve patients’ quality of life after liver transplantation. Future studies should consider age and time since transplantation in mental health analyses. The lack of a mediating or moderating effect on resilience between PTG and the TxEQ-Spanish opens new avenues for psychological research. Future studies should explore additional contributing variables (Supplementary File S2).

The current cross-sectional design offers valuable insights; however, a longitudinal approach could deepen our understanding of the dynamic relationships between resilience, PTG, and treatment adherence. Tracking these variables over time, alongside life events, stressors, and evolving perceptions of the transplant experience, would provide a more comprehensive view of their interplay. Such research could better elucidate the psychological and emotional trajectories of LT patients.

Our study has limitations that warrant consideration. The use of self-administered questionnaires may have introduced bias due to subjective interpretations or limited comprehension of specific questions. The single-center nature of this study restricts the generalizability of our findings to broader contexts or demographics. Additionally, the lower proportion of women in our sample reflects the demographic profile of our center’s LT population, although it limits the strength of gender-based comparisons. Future studies should adopt multicenter designs to enhance sample size and diversity, implement stratified sampling for balanced gender representation, and consider oversampling women to allow for more robust gender comparisons. Data collection during May–July 2021 coincided with the COVID-19 pandemic, which likely influenced resilience and adherence outcomes due to heightened stress, disrupted healthcare services, and altered daily routines.

This study highlights the need to tailor approaches to liver transplant candidates to improve outcomes. It suggests that the emotional attitudes of both men and women should be considered in the program, as women’s post-traumatic growth is seen to be greater with greater use of social support, while men’s growth is focused on enhancing personal strength and motivation. To account for these differences, unique nursing strategies can be created, such as developing family-centered support systems for women and developing self-efficacy for men. In addition to this, addressing mental health issues such as guilt or worry and secrecy issues related to transplantation can greatly improve the quality of life of these patients. The delivery of these services by a multidisciplinary team of professionals such as psychologists and social workers is important and critical. These strategies not only aim to improve the psychological state of patients but also to facilitate treatment adherence and mitigate complications, which ultimately influence the outcome after liver transplantation.

## 5. Conclusions

There are significant differences between men and women in terms of post-traumatic growth following liver transplantation. Furthermore, the level of resilience is correlated differently between men and women. Resilience in men is correlated with personal strength, whereas resilience in women is correlated with relationships with others. Resilience does not have a mediator or modulate effect in PTG and the TxEQ-Spanish. These differences will help us to better understand the psychological events suffered by patients throughout the course of follow-up.

The results provide us with the requisite knowledge to validate and enhance our intervention with the patient’s gender perspective in this process. It is recommended that nursing interventions be tailored to the specific needs of men and women. To enhance resilience in men, it is essential to concentrate on specific personality traits, motivations, capabilities, and self-esteem. Conversely, in women, interventions should focus on their family and social relationships. It is imperative that a gender perspective be incorporated into nursing interventions in order to enhance our understanding of the factors that contribute to emotional fluctuations and to develop effective strategies for their management. Future studies should use a mixed methodology (quantitative–qualitative) that expands the information on gender differences and roles in society.

## Figures and Tables

**Table 1:** Sociodemographic data.

	Men (*n* = 132) Md [IQR]	Women (*n* = 42) Md [IQR]	*p* Value
Age at LT	53 [18–68]	55.50 [21–70]	0.376
Years from LT	8.50 [0–38]	8 [1–29]	0.927
Age at study	62 [25–86]	65.50 [24–83]	0.728

LT: liver transplantation; Mann–Whitney U test.

**Table 2:** Impact of liver transplantation with a gender perspective.

Connor–Davidson 10 Resilience Scale (CD-RISC)				
Variable Md [IQR]	Total (*n* = 174)	Male (*n* = 132)	Female (*n* = 42)	*p* Value
**Resilience**	32 [27–36]	32 [27–36]	32 [27–35]	0.525
**Post-Traumatic Growth Inventory (PTGI 21-item)**				
Variable Md [IQR]	**Total (*n* = 174)**	**Male (*n* = 132)**	**Female (*n* = 42)**	***p* Value**
New Possibilities	3.71 [2.86–4.29]	3.57 [2.86–4.25]	3.86 [3–4.71]	0.062
Relating to Others	3.4 [2.4–4]	3.3 [2.4–4]	3.6 [2.8–4.6]	0.088
Personal Strength	3.75 [2.75–4.5]	3.5 [2.75–4.25]	4.25 [3.25–4.75]	0.010 *
Spiritual Change	3 [1–4]	3 [1–4]	4 [2–5]	0.010 *
Appreciation of Life	4 [3.33–4.67]	4 [3.33–4.67]	4.33 [3.67–4.75]	0.168
**Transplant Effects Questionnaire Spanish (TxEQ-Spanish 22-item)**				
**Variable Md [IQR]**	**Total (*n* = 174)**	**Male (*n* = 132)**	**Female (*n* = 42)**	***p* Value**
Worry	3.33 [2.83–3.83]	3.33 [3–3.83]	3.25 [2.67–3.87]	0.505
Guilt	1.875 [1.25–2.5]	2 [1.25–2.5]	1.75 [1.19–2.5]	0.935
Disclosure	4.67 [3.67–5]	4.67 [3.67–5]	5 [3.67–5]	0.289
Responsibility	4 [3.5–5]	4.25 [3.5–5]	4 [3.5–5]	0.796
Adherence	4.6 [3.8–5]	4.8 [3.65–5]	4.6 [3.8–5]	0.819

Mann–Whitney U test; * *p* < 0.05 statistically significant.

**Table 3:** Resilience correlation with age, time from liver transplantation, and TxEQ-Spanish and PTGI questionnaires (*n* = 174).

Connor–Davidson 10 Resilience Scale		
*n* = 174	Pearson Correlation	*p* Value
Age	−0.071	0.352
Month from Liver Transplant	−0.028	0.717
**Post-Traumatic Growth Inventory**		
New Possibilities	0.312 **	0.000
Relating to Others	0.400 **	0.000
Personal Strength	0.430 **	0.000
Spiritual Change	0.239 **	0.001
Appreciation of Life	0.303 **	0.000
**Transplant Effects Questionnaire Spanish**		
Worry	−0.065	0.395
Guilt	−0.025	0.742
Disclosure	0.129	0.089
Responsibility	0.100	0.187
Adherence	0.157 *	0.039

* Correlation is significant at the 0.05 level (bilateral)/** correlation is significant at the 0.01 level (bilateral).

**Table 4:** Multiple linear regression analysis by gender: factors associated with resilience.

Independent Variable	Male Gender			
	Regression Coefficient (β)	Standard Error	(95% Conf. Intervals)	*p* *
PTGI				
Relating to Others	0.791	0.717	(−0.628–2.211)	0.272
Personal Strength	2.233	0.861	(−0.528–3.938)	0.011 *
Appreciation of Life	−0.876	0.976	(−2.809–1.056)	0.371
TxEQ-Spanish				
Adherence	1.034	0.614	(−0.180–2.250)	0.094
Cons	19.651	3.728	(12.273–27.029)	0.000
	**Female Gender**			
	**Regression Coefficient (β)**	**Standard Error**	**(95% Conf. Intervals)**	*p* *
PTGI				
Relating to Others	3.279	1.132	(0.985–5.574)	0.006 *
Personal Strength	1.088	1.294	(−0.1.533–3.710)	0.406
Appreciation of Life	−0.953	0.971	(−4.376–2.469)	0.576
TxEQ-Spanish				
Adherence	1.923	0.971	(−0.045–3.891)	0.055
Cons	9.737	7.595	(−5.653–25.127)	0.208

TxEQ-Spanish: Transplant Effects Questionnaire Spanish; PTGI: Post-Traumatic Growth Inventory. * Statistically significant (*p* < 0.05).

**Table 5:** Results of the mediation analysis of resilience.

Effect	Route	Coefficient	SE	95% CI	
				LS	LI
Direct effect of PTG on resilience	a	0.481 ***	0.086	0.650	0.312
Direct effect of resilience in TxEQ-Spanish	b	0.018	0.025	0.067	−0.031
Direct effect of PTG in TXEQ-Spanish	c’	0.086 *	0.031	0.147	0.025
Total effect of PTG in TxEQ-Spanish	c	0.095 **	0.028	0.150	0.040
Indirect effect	ab	0.009	0.012	0.033	−0.015
Sobel test	z	0.009	0.012	0.650	0.312
Total effect of the TxEQ model (R^2^ = 0.081)					

Coefficient: unstandardized beta coefficient; SE: standard error; 95% CI: 95% confidence interval; * *p* < 0.05; ** *p* < 0.01; *** *p* < 0.000.

## Data Availability

Data are available upon contact with and authorization from the authors of this study.

## Supplementary Materials

The following supporting information can be downloaded at: https://www.mdpi.com/article/10.3390/healthcare13010024/s1, Supplementary File S1: Methodology employed in the scoring of the questionnaires Supplementary File S2: A comparison of the results of the present study and previous findings.
