# Supplementary material for: Resilience, Post-Traumatic Growth, and Transplant Effects—Gender Differences Following Liver Transplantation: A Cross-Sectional Study
Source: Healthcare (Basel). 2024 Dec 26;13(1):24. doi: 10.3390/healthcare13010024 (PMC11720365; doi:10.3390/healthcare13010024)
Supplement: Supplementary file 1 [file healthcare-13-00024-s001.zip › healthcare-3352718-supplementary.pdf]

Supplementary File S1: Methodology employed in the scoring of the questionnaires.

-Connor-Davidson Resilience Scale (CD-RISC)

The CD-RISC is a 10-item scale that measures resilience. Each item is rated on a 5-point scale, where 0 means "not true at all" and 4 means "true nearly all the time." The total score ranges from 0 to 40, with a higher score indicating greater resilience.

-21-item Post Traumatic Growth Inventory (PTGI) scale

The PTGI assesses post-traumatic growth through 21 items grouped into five factors: Personal Strength, New Possibilities, Relating to Others, Spiritual Change, and Appreciation of Life. Each item is rated on a 6-point scale, where 0 means "I did not experience this as a result of my crisis" and 5 means "I experienced this to a very great degree as a result of my crisis." The total score ranges from 0 to 105, with a higher score indicating greater post-traumatic growth.

-Transplant Effects Questionnaire (TxEQ-Spanish)

The Transplant Effects Questionnaire (TxEQ) is a tool designed to evaluate the psychological and emotional effects associated with organ transplantation. The Spanish version (TxEQ-Spanish) was validated among liver transplant recipients. The scale evaluates five main factors: transplant-related concerns, guilt regarding the donor, disclosure of the transplant, adherence to treatment, and responsibility towards the donor, family, friends, or medical staff. Each item is rated on a 5-point scale, where 1 means "I strongly agree" and 5 means "I strongly disagree". For the overall score of the TxEQ questionnaire, a sum of the scores of the 5 dimensions was made.

Supplementary File S2: A comparison of the results of the present study and previous findings

Study results	Previous findings
LT women have higher scores in all dimensions of PTG, being statistically significant in "relationship with others" and "spiritual changes".<br><br>PTG is significant in the resilience of these patients, behaving differently in men than in women.	No significant differences in the gender subgroup; Pérez-San Gregorio et al.,2017 [8]
No significant differences in the connection of time since transplantation, and PTG.	Similar results to Pérez-San Gregorio et al., (2017) [8]
Women have a higher score than men in all dimensions, so we could assume that they have more effective adaptive coping strategies.<br>Women present differences compared to men and therefore strategies and intervention are required from a gender perspective.	Differences are shown between men and women in coping styles, appraisal of stress transplantation, and coping resources in the long-term; Grady et al., 2016 [32]<br><br>LT patients had a better adaptive coping strategy associated with greater PTG; Pérez-San-Gregorio et al., (2017) [11].<br><br>LT men had the need to increase dignity and their significant family position, on the contrary, positive experiences from other transplant recipients and more psychological support were required by women; Chen et al., 2021 [21].
No gender differences in resilience after liver transplantation, nor in relation to time since liver transplantation was described.<br><br>Our results describe the correlation of resilience with all dimensions of post-traumatic growth and adherence to treatment.	Resilience's pathways are not concordant between sexes; Fallon et al. (2020) [34].<br><br>Resilience positively correlates with PTG in cadaveric kidney transplant recipients. Resilience is described as a possible predictor of PTG; Tomaszek et al. (2021) [33].
Negative correlation of resilience with worry and guilt. Adherence to treatment has a significant weight on the level of resilience, especially in women.	A recent study in young adult transplant recipients describes a gender difference in therapeutic adherence; Vaisbourd et al., 2023 [36].<br><br>Guilt, responsibility and worries are related to limited mental health, whereas a higher mental health was associated with disclosure about transplantation; Scheel et al., 2019 [38].
